# BET Inhibition Synergizes with RLR Signaling to Enhance Tumor Immunogenicity and T-cell Recognition

**DOI:** 10.1158/2326-6066.CIR-25-0777

**Published:** 2026-07-09

**Authors:** Lucas Baldran-Groves, Rok Žiberna, Marc-Antoine Gerault, Mireia Cruz De los Santos, Adela Kiessling, Sylvya Pasca, Nooshin Khoshdoozmasouleh, Andreas Post, Andreas Lundqvist, Stina Wickström, Rolf Kiessling, Jeroen Melief

**Affiliations:** 1 Department of Oncology-Pathology, Karolinska Institutet, Stockholm, Sweden.; 2 Theme Cancer, Patient Area Head and Neck, Lung and Skin, Karolinska University Hospital, Stockholm, Sweden.; 3 Department of Clinical Neuroscience, Karolinska Institutet, Stockholm, Sweden.

## Abstract

Intratumoral signaling via the cytosolic double-stranded RNA (dsRNA) receptors RIG-I and MDA-5 enhances antitumor immunity and can overcome resistance to cancer immunotherapy. Although bromodomain extraterminal (BET) proteins are established epigenetic drivers of immune escape mechanisms, their role in regulating RIG-I–like receptor (RLR) signaling remains undefined. Therefore, we investigated the role of BET proteins in modulating innate antiviral responses to cytosolic dsRNA in tumor cells and how BET inhibition combined with dsRNA stimulation influences tumor immunogenicity and recognition by T cells. To address this, human tumor cell lines were treated with the BET inhibitor (BETi) JQ1 and transfected with the synthetic viral dsRNA analog poly(I:C) (pIC) to stimulate innate antiviral signaling. We found that BET inhibition synergistically amplifies dsRNA-induced immunogenicity, as indicated by enhanced cytokine and chemokine production, JAK–STAT signaling, antigen presentation, and immunogenic cell death. Combined treatment of melanoma cells with JQ1 and pIC resulted in a marked, synergistic enhancement of recognition by autologous tumor-specific CD8^+^ tumor-infiltrating lymphocytes and maturation of dendritic cells. Furthermore, an antiviral gene signature induced by pIC and enhanced by JQ1 correlated with immune infiltration and improved survival in larger patient cohorts across different types of cancer. Overall, our findings demonstrate that targeting BET proteins in tumor cells strongly potentiates dsRNA-induced antiviral responses and suggest that combining BETis with RLR agonists offers a pharmacologic strategy to enhance antitumor T-cell responses and improve the clinical efficacy of cancer immunotherapies.

## Teaser

The authors report that BET inhibitors can improve antitumor T-cell responses by amplifying dsRNA-induced innate antiviral signaling, a finding that could be exploited to enhance the efficacy of cancer immunotherapies, particularly in treatment-refractory patients.

## Introduction

Immunotherapies that harness autologous tumor-specific T cells, including immune checkpoint inhibition and adoptive cell transfer of *ex vivo*–expanded tumor-infiltrating lymphocytes (TIL), have led to unprecedented improvements in clinical outcomes for patients with late-stage cancer (1). However, many patients still fail to derive durable benefits from these therapies due to tumor-intrinsic immune evasion mechanisms, including defects in interferon (IFN) signaling and disruption of components of the HLA class I (HLA-I) antigen processing and presentation machinery (HLA-I APM), which impair recognition of tumor-specific epitopes presented to CD8^+^ T cells (2). For instance, melanoma lesions often harbor loss-of-function mutations in genes encoding components of the HLA-I APM and/or JAK–STAT signaling pathways (3).

In addition to genetic alterations, increasing evidence indicates that epigenetic mechanisms can drive immune escape by shaping tumor–immune interactions and therefore represent promising targets for therapeutic intervention (4). In this regard, bromodomain (BRD) extraterminal (BET) proteins constitute an important class of epigenetic regulators with emerging relevance to antitumoral T-cell responses. Physiologically, BET proteins act as epigenetic “readers” that use BRD modules to bind acetylated lysines on histone tails, thereby regulating genes involved in proliferation and survival, including *MYC* (5). Although initially developed as antiproliferative drugs, BET inhibitors (BETi) have been shown to modulate tumor immunity, for instance, by downregulating PD-L1 or enhancing HLA-I expression (6, 7).

Tumor-intrinsic detection of double-stranded RNA (dsRNA) via the RIG-I–like receptors (RLR) RIG-I and MDA-5 is increasingly recognized as critical for effective antitumor immunity. Upon binding of endogenous or viral dsRNA species, these cytosolic receptors activate innate antiviral programs characterized by type I/III IFN production and proinflammatory cytokines (8). Beyond antiviral defense, intact RIG-I signaling in tumor cells is crucial for response to checkpoint blockade and can overcome immune evasion mechanisms by promoting antitumor T-cell immunity through both IFN-dependent and independent pathways (9).

Here, we investigated how BET inhibition influences dsRNA-induced signaling and its downstream effects on tumor immunogenicity and functional antitumor T-cell responses *in vitro*. We demonstrated that prolonged BET inhibition markedly sensitized tumor cells for activation of the RIG-I/MDA-5 signaling pathway. Enhanced dsRNA-induced signaling resulted in increased production of type I and III IFNs and chemokines, boosted HLA-I–restricted antigen presentation, and enhanced immunogenic cell death (ICD) in tumor cells. Treated tumor cells more effectively activated both tumor-associated and neoantigen-specific CD8^+^ T cells and enhanced the maturation of dendritic cells (DC), indicating broad immunogenic effects. Finally, the antiviral gene signature amplified by BETi negatively correlated with a tumor-intrinsic immune resistance signature and positively correlated with immune infiltration and overall survival (OS) in larger cohorts of (immunotherapy-treated) patients.

## Materials and Methods

### Sex as a biological variable

Both male and female subjects were included. Sex was not analyzed as an independent biological variable.

### Culture of tumor cell lines and TILs

Cell lines and primary material were collected over the period 2011 to 2026 and cryopreserved in liquid nitrogen for long-term storage. The A375 melanoma (RRID: CVCL_0132) and COLO320 (RRID: CVCL_1989) colorectal cancer cell lines were purchased from the American Type Culture Collection. A375, A375_VR4_, and COLO320 cells were cultured in RPMI 1640 (Gibco, cat. #11875093) with 10% fetal bovine serum (FBS; Gibco, cat. #A5256701), 1% penicillin–streptomycin (Gibco, cat. #15140122), and 5 µg/mL Plasmocin (InvivoGen, cat. #ant-mpt). ANRU, KADA, and BEHA tumor cell lines were established from three patients with melanoma at the oncology clinic at Karolinska University Hospital (ethical permit: #2011/143-32/1 and 2015/1862–32) and cultured in Iscove’s modified Dulbecco’s medium (Gibco, cat. #12440053) or RPMI 1640 with 10% to 20% FBS, 1% penicillin–streptomycin, and 5 µg/mL Plasmocin. TILs from these same patients were isolated, cultured, and expanded as previously described (10). Cell lines and TILs were not tested for *Mycoplasma* contamination. The study was conducted in accordance with institutional and national ethical guidelines and the principles of the Declaration of Helsinki. Written informed consent was obtained from all participants, and the study protocol was approved by the relevant ethics review board. Additional information on cell lines is listed in Supplementary Table S1.

### Drug treatment

For 72 hours of treatment, tumor cells were seeded on flat-bottom six-well plates (Sigma-Aldrich, cat. #Z707759) at a density of 20,000 to 300,000 cells/well, based on growth rates and sensitivity to treatment for adequate transfection with pIC when wells were 70% to 90% confluent. Adherent cells were treated 24 hours after seeding with the BETi JQ1 (Tocris Bioscience, cat. #4499, 0.4 µmol/L), I-BET151 (Tocris Bioscience, cat. #4650, 0.4 µmol/L), or dBET1 (MedChemExpress, cat. #HY-101838, 0.4–1 µmol/L) for 72 hours, unless stated otherwise. In experiments using direct MYC inhibitors, cells were treated for 24 hours with MYCMI-6 (MedChemExpress, cat. #HY-124675, 3–5 µmol/L) or for 48 hours with MYC-RIBOTAC (MedChemExpress, cat. #HY-153713, 3–5 µmol/L). In experiments with IFNγ, cells were treated simultaneously with JQ1 (0.4 µmol/L) and/or recombinant human IFNγ (PeproTech, cat. #300-02, 25 ng/mL) for up to 72 hours. An equal amount of DMSO (Sigma-Aldrich, cat. #D2650) was added to the untreated cells as a control. Additional information on reagents used is listed in Supplementary Table S1.

### RNA interference experiments

Targeted gene silencing of TLR3 (cat. #4390824), RIG-I (cat. #4392420), MDA-5 (cat. #4392420), ADAR (cat. #4390824), and NLRX1 (cat. #4392420) was done using Silencer Select siRNAs (Thermo Fisher Scientific). Silencer RNAs or nontargeting control siRNAs (cat. #4390843) were transfected using Lipofectamine RNAiMAX (Thermo Fisher Scientific, cat. #13778150) according to the manufacturer’s protocol. Briefly, 1 × 10^5^ tumor cells were seeded in six-well plates and left overnight to adhere. Cells were then transfected with targeting siRNAs or nontargeting control siRNAs (25 nmol/L) for 72 hours before downstream analysis or pIC transfection. Additional information on siRNAs is listed in Supplementary Table S2.

### Stimulation with pattern recognition receptor agonists

Intracellular delivery of pattern recognition receptor (PRR) agonists (InvivoGen) was carried out using Lipofectamine 2000 (Thermo Fisher Scientific, cat. #11668027) according to the manufacturer’s instructions. Briefly, a transfection mix was prepared in Opti-MEM with Lipofectamine 2000 (10 µg/mL) complexed with the following reagents: pIC (High Molecular Weight; 1 µg/mL, cat. #tlrl-pic), imiquimod (5 µg/mL, cat. #tlrl-imqs-1), ODN 2006 (1 µmol/L, cat. #tlrl-2006), or 2′3′-cGAMP (1 µg/mL, cat. #tlrl-nacga23-02). After DMSO/JQ1 pretreatment, the culture medium was replaced with 500 μL of transfection medium per well in a six-well plate for 4 hours, after which it was replaced with fresh culture medium (2 mL) and incubated for an additional 24 to 48 hours. Untransfected pIC (pIC only, 1 µg/mL), Pam3CSK4 (10 ng/mL, cat. #tlrl-pms), FSL-1 (100 ng/mL, cat. #tlrl-fsl), LPS-EK (1 µg/mL, cat. #tlrl-eklps), FLA-ST (100 ng/mL, cat. #tlrl-stfla), ssRNA40/LyoVec (1 µg/mL, cat. #tlrl-lrna40), or 3pRNA/Lyovec (1 µg/mL, cat. #tlrl-3prnalv) was directly added to the cells upon fresh media replacement. The IFNα/βR1 blocking antibody (2 µg/mL, R&D Systems, cat. #AF245) or JAK inhibitor ruxolitinib (2 µmol/L, RayBiotech, cat. #331-10377-3) were added to the cells upon fresh media replacement.

### Quantitative real-time PCR

For gene expression analysis by quantitative real-time PCR (qPCR or qRT-PCR), treated tumor cells were washed with PBS and lysed using TRIzol reagent (Thermo Fisher Scientific, cat. #15596026). Total RNA was purified using the PureLink RNA Mini Kit (Thermo Fisher Scientific, cat. #12183025) according to the manufacturer’s instructions. The quality and concentration of the purified RNA were verified using NanoDrop2000 (Thermo Fisher Scientific, cat. #ND-2000). RNA (500 ng) was used to generate complementary DNA using the iScript cDNA Synthesis Kit (Bio-Rad, cat. #1708890) and stored at −20°C until further use.

qRT-PCR was performed using iTaq Universal SYBR Green Supermix (Bio-Rad, cat. #1725121) on a QuantStudio7 Flex Real-Time PCR System (Thermo Fisher Scientific) according to the manufacturer’s instructions. Each reaction was performed in triplicate. Relative gene expression was calculated using the ΔΔCT method. The transcript levels of glyceraldehyde 3-phosphate dehydrogenase (GAPDH) were used for normalization. Primer sequences used in this study are listed in Supplementary Table S3.

### Western blot

A375 cells were pretreated with DMSO or JQ1 and subsequently transfected with pIC as described. After 24 hours of pIC stimulation, cells were lysed in IP lysis buffer supplemented with a protease inhibitor and centrifuged, after which protein concentrations were quantified using the Pierce BCA Protein Assay Kit (Thermo Fisher Scientific) according to the manufacturer’s instructions. Protein samples were separated by SDS-PAGE, transferred to nitrocellulose membranes, and blocked in 5% bovine serum albumin (BSA; Sigma-Aldrich, cat. #A7030), followed by overnight incubation in primary antibody at 4°C. Antibodies used for Western blot are indicated in Supplementary Table S4. The membranes were incubated with a secondary antibody, either horseradish peroxidase (HRP)–conjugated [goat anti-rabbit IgG (H+L), Thermo Fisher Scientific, cat. #31460] and detected with ECL substrate using an iBright Imaging System or IRDye-conjugated (IRDye 680RD goat anti-mouse IgG, LICORbio, cat. #926-68070) and detected with a LICORbio Odyssey System. Western blot bands were quantified using the Quantity One program (Bio-Rad Laboratories Inc.), subtracting the background level and normalizing to the loading protein (GAPDH) detected on the same membrane across multiple experiments.

### Quantification of tumor-derived cytokines

CXCL10, IFNβ, and IFNλ1 concentrations of melanoma cell line supernatants were determined using the following ELISA kits according to the manufacturer’s instructions: human CXCL10/IP-10 DuoSet ELISA (R&D Systems, cat. #DY266), human IFN-beta DuoSet ELISA (R&D Systems, cat. #DY814-05), and human IL-29/IFN-lambda 1 DuoSet ELISA (R&D Systems, cat. #DY7246). Wherever indicated, cytokine concentrations were normalized to tumor cell numbers after treatment to account for toxicity.

### Flow cytometry

All antibodies and reagents were used according to the manufacturer’s instructions unless stated otherwise. Antibodies used for flow cytometry are listed in Supplementary Table S4. Cells were plated on a 96-well V-bottom plate (Costar) with 0.8 to 1.5 × 10^5^ cells per well. Cells were then washed with PBS and incubated with a dead cell marker (LIVE/DEAD Fixable Aqua Dead Cell Stain Kit, Thermo Fisher Scientific, cat. #L34957) for 15 minutes at room temperature. For extracellular staining, antibodies were diluted in FACS buffer (PBS + 1% BSA) and incubated for 30 minutes at 4°C in the dark. For intracellular staining, cells were washed with PBS and fixed with the BD Cytofix/Cytoperm kit (BD Biosciences, cat. #554714) or with the eBioscience Foxp3/Transcription Factor Staining Buffer Set (Thermo Fisher Scientific, cat. #00-5523-00). The fixed cells were washed with kit buffers and stained for 30 to 40 minutes at 4°C in the dark. Cells were then resuspended in FACS buffer, and samples were run on a NovoCyte (ACEA Biosciences). Compensation matrices were generated from compensation beads (AbC Total Compensation Beads, cat. #A10497, and ArC Reactive Beads, cat. #A10628, Invitrogen), according to the manufacturer’s protocol. Flow cytometry data were analyzed using the FlowJo software (RRID: SCR_008520, version 10.8.1, TreeStar Inc.). Cells were gated as indicated in Supplementary Fig. S1.

### RNA sequencing: sample preparation

ANRU tumor cells were treated as previously described. To account for noise generated by the transfection procedure, untransfected cells were treated with a similar concentration (10 µg/mL) of Lipofectamine 2000 without RNA. Three biological replicates were included for each treatment group. RNA from the treated tumor cells was extracted and purified using the PureLink RNA Mini Kit (Thermo Fisher Scientific) according to the manufacturer’s instructions. RNA integrity was assessed using the Bioanalyzer 2100 system (Agilent Technologies). Messenger RNA was purified from the total RNA using poly T oligo–attached magnetic beads. After fragmentation, the first-strand cDNA was synthesized using random hexamer primers. Next, second-strand cDNA was synthesized using dUTP. The directional library was prepared after end repair, A-tailing, adapter ligation, size selection, amplification, and purification. The library was checked with Qubit and real-time PCR for quantification and a bioanalyzer for size distribution detection. After library quality control, different libraries were pooled based on the effective concentration and targeted data amount and then subjected to Illumina HiSeq 4000 (Novogene).

### RNA sequencing: data analysis

The reference genome and gene model annotation files were downloaded directly from the genome website. The index of the reference genome was built using HISAT2 (RRID: SCR_015530, v2.0.5), and paired-end clean 1 reads were aligned to the reference genome. featureCounts (RRID: SCR_012919, v1.5.0-p3) was used to count the read numbers mapped to each gene, and fragments per kilobase of sequence per million mapped reads was calculated based on the length of the gene and the read count mapped to this gene. Differential expression analysis for two conditions was performed using the DESeq2 R package (RRID: SCR_015687, v1.20.0). The resulting *P* value was adjusted using the Benjamini and Hochberg method to control the FDR. The adjusted *P* value ≤0.05 and |log_2_(fold change)| ≥ 1 were set as the thresholds for significant differential expression. Overrepresentation analysis of differentially expressed genes was performed using the clusterProfiler R package (RRID: SCR_016884) with the Gene Ontology (GO) biological process database. Terms with adjusted *P* values less than 0.05 were considered significantly enriched in differentially expressed genes. For gene set enrichment analysis (GSEA), we used the local version of the GSEA tool (http://www.broadinstitute.org/gsea/index.jsp, RRID: SCR_003199). Protein-protein interaction networks were performed in Cytoscape (RRID: SCR_003032, v3.10.3) with the stringApp. Gene signatures used in this study are available in Supplementary Table S5.

### Tumor–TIL coculture

Treated KADA, ANRU, and BEHA tumor cells were plated on a 96-well U-bottom sterile plate (40,000 cells/well). Where indicated, HLA-I was blocked by preincubating tumor cells with 10 μg/mL Ultra-LEAF Purified HLA-A,B,C antibody (BioLegend, cat. #311428) for 60 minutes at 37°C before the addition of TIL (TIL/tumor ratio = 5:1). TILs only or TILs incubated with CD3/CD28 activating Dynabeads (Gibco, cat. #10310614) were used as negative and positive controls, respectively. For flow cytometry readout, TILs were incubated with a lysosomal-associated protein 1 (LAMP1, CD107a) antibody for 2 hours. Then, BD GolgiStop (BD Biosciences, cat. #554724) and BD GolgiPlug (BD Biosciences, cat. #555029) were mixed (1:1.5) and added to the wells for another 3 hours at 37°C. Finally, the cells were transferred to a 96-well V-bottom plate (Costar) and stained for extracellular or intracellular markers. For experiments using dextramers, ANRU TILs were incubated at room temperature for 10 minutes with HLA-A*02:01/peptide-specific dextramers for MART-1 or mutant NUP210 (Immudex) prior to the addition of extracellular antibodies, as previously described (11). For IFNγ ELISA as a readout, tumor cells and TILs were cocultured for 24 hours before the transfer of the supernatant at −20°C and stored for subsequent IFNγ ELISA.

### IFNγ ELISA

Sandwich ELSA was performed to detect secreted IFNγ in the supernatant of the tumor–TIL coculture plate. IFNγ ELISAs [ELISA Flex: Human IFN-γ (HRP), Mabtech, cat. #3420-1H] were performed according to the manufacturer’s protocol.

### Generation and maturation of DCs

Peripheral blood samples from healthy donors were purchased from the Karolinska University Hospital Blood Bank (ethical permit #2024/07830/01). All donors provided informed consent for the research use of their samples, and all procedures were conducted in accordance with the Declaration of Helsinki. Peripheral blood mononuclear cells (PBMC) were isolated from healthy donor buffy coats by density centrifugation with Ficoll-Paque Plus (GE HealthCare, cat. #GE17-1440). CD14^+^ monocytes were isolated from PBMCs using CD14^+^ microbeads (Miltenyi Biotec, cat. #130-097-052), following the manufacturer’s instructions.

To generate immature DCs (iDC), CD14^+^ monocytes were cultured in CellGro (CellGenix, cat. #20805-0500) supplemented with IL4 (20 ng/mL, PeproTech, cat. #200-04) and granulocyte-macrophage colony-stimulating factor (100 ng/mL, CellGenix, cat. #1412-050) for 48 hours. iDCs were then harvested, counted, and seeded in 12-well plates (1 × 10^6^ cells in 1 mL CellGro). Treated A375 cells were then added to iDCs (iDC/A375 = 3:1). In experiments with tumor-derived media, supernatants from treated A375 cells were collected, centrifuged (700 *g*, 5 minutes), and added to iDCs (CellGro: A375 SN = 1:3). Cells were incubated for an additional 48 hours at 37°C before harvesting for flow cytometry. In conditions where iDCs were cocultured with A375 cells, stained iDCs were gated based on the expression of CD80 and CD86 for flow cytometry analysis (see Supplementary Fig. S1: gating strategy).

### Jerby-Arnon and colleagues mapping analysis

The data and code used by Jerby-Arnon and colleagues were obtained from https://github.com/livnatje/ImmuneResistance. The top hundred differentially expressed genes, according to the adjusted *P* value from the selected treatment comparison quantified in cohorts from Jerby-Arnon and colleagues, were used to generate gene signatures for their respective bulk RNA sequencing (RNA-seq) data from The Cancer Genome Atlas (TCGA) cohort of 473 skin cutaneous melanoma tumor samples (12). Gene signatures were computed as described by Jerby-Arnon and colleagues using the function *compute.samples.res.scores* from their GitHub repository. Survival analysis was performed using the survival R package. Additional correlation and survival analyses were performed using GEPIA 2 (http://gepia2.cancer-pku.cn/) and Kaplan–Meier plotter (https://kmplot.com/analysis/).

### Synergy analysis

Synergy between JQ1 and poly(I:C) (pIC) was assessed using the Bliss independence model. Raw experimental values were used for the analysis of CXCL10 and IFNγ secretion, except for HLA-I expression data, which were log_10_-transformed prior to analysis.

For each experiment, Bliss independence values (“Bliss JQ1 + pIC”) were calculated separately for each cell type and biological replicate according to

$$E_{Bliss}=E_{JQ1}+E_{pIC}-\left(E_{JQ1}\times E_{pIC}\right)$$

where $E_{JQ1}$ and $E_{pIC}$ represent the effects of JQ1 and pIC administered individually.

The observed effect of the combination treatment (JQ1 + pIC) was compared with the corresponding Bliss-predicted value. The Bliss difference (“Bliss_diff”) was defined as the mean difference between the measured combination effect and the theoretical effect predicted by the Bliss independence model. Positive Bliss difference values indicate synergy.

For each cell type within each experiment, statistical significance was assessed using a Student *t* test comparing the observed combination effect with the Bliss-predicted effect. *P* values were calculated independently for each cell type and experiment. Additional information can be found in Supplementary Table S6.

Using this approach, JQ1 and pIC demonstrated significant synergistic effects across different cell types in all three experiments tested.

### Statistical analysis

Statistical analysis and graphical layout were performed using GraphPad Prism software (RRID: SCR_002798, v10.4.1) or in R. Data in bar plots are presented as the mean ±SD of biological replicates unless stated otherwise. For statistical analysis of group comparisons, repeated measures-paired one- or two-way ANOVA was performed. If the ANOVA test was significant, Holm–Šídák multiple comparison tests were performed. *P* values are indicated atop comparisons in graphical layouts or annotated accordingly: *, *P* < 0.05; **, *P* < 0.01; ***, *P* < 0.001; ****, *P* < 0.0001.

### Study approval

All patient-derived materials and PBMCs were obtained with written informed consent under approval from the regional ethics committee (Dno. 2011/143–32/1, 2015/1862–32, and 2024/07830/01) in accordance with the Declaration of Helsinki.

### Supplementary material

Supplementary information on reagents used in this study can be found in Supplementary Tables S1 to S4. Gene signatures used in this study are displayed in Supplementary Table S5. Synergy analysis can be found in Supplementary Table S6. Supplementary Figures S1 to S13 are available online.

## Results

### BETi pretreatment sensitizes tumor cells to RLR-induced inflammation

**Figure 1.**
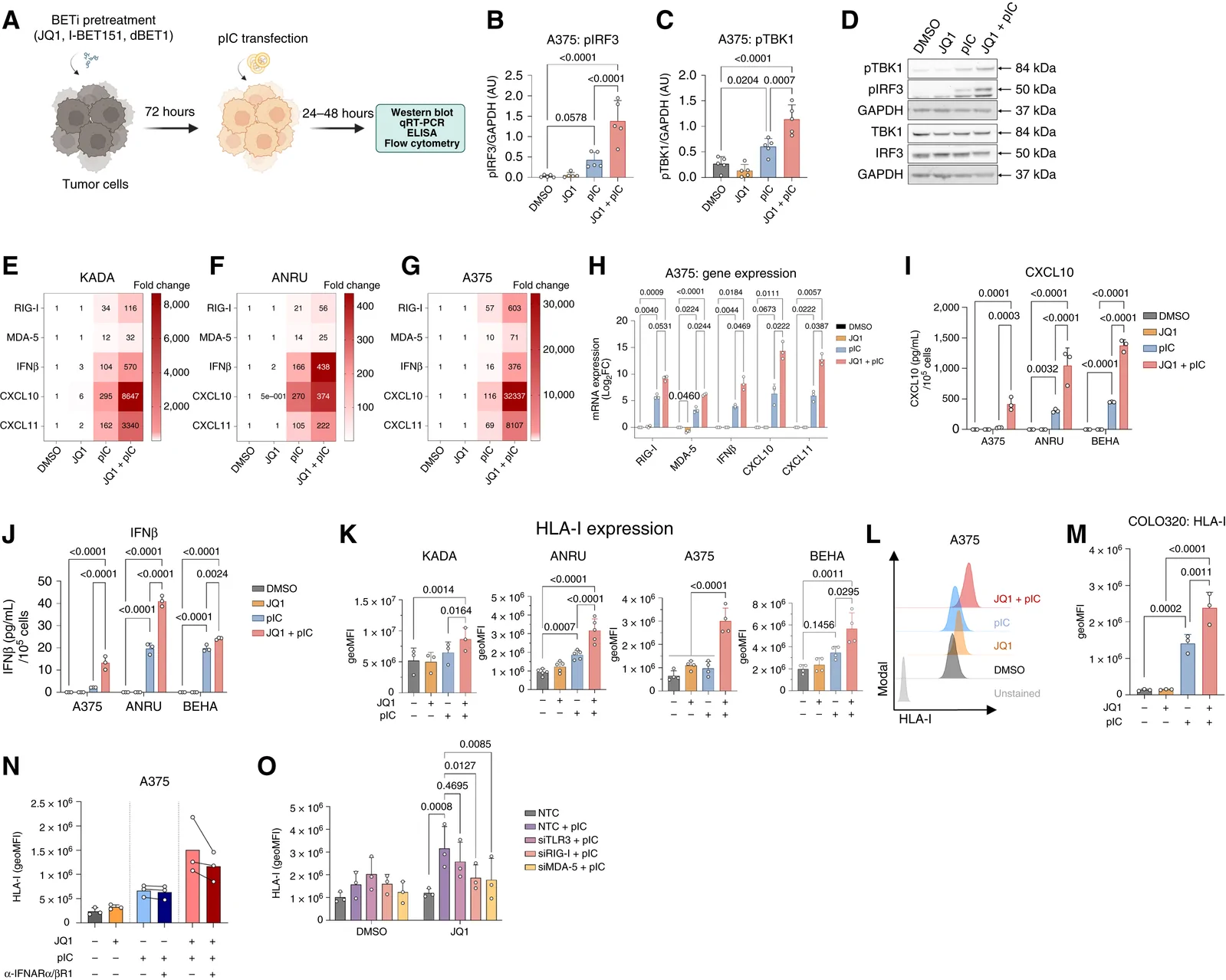
BETi pretreatment sensitizes tumor cells to pIC-induced inflammation. **A,** Experimental setup for BETi pretreatment and pIC transfection of tumor cells. **B,** Protein abundance of phosphorylated IRF3 and (**C**) TBK1 in A375 cells after 72 hours of JQ1 or DMSO pretreatment and pIC transfection for 24 hours assessed by Western blot. *n* = 5 biological replicates. A representative Western blot is depicted in (**D**). **E,** Expression heatmap of selected genes from qPCR after 72 hours of JQ1 treatment and subsequent pIC transfection for 24 hours in ANRU, (**F**) KADA, and (**G**) A375. Data are represented as mean fold change from DMSO. *n* = 3 biological replicates/cell line. **H,** Bar plot displaying gene expression of selected genes from **G** (log_2_ fold change from DMSO). **I,** CXCL10 and (**J**) IFNβ concentration in supernatants from treated A375, ANRU, and BEHA cells. *n* = 3 biological replicates/cell lines. **K,** Cell surface expression of HLA-I was assessed by flow cytometry after 48 hours of pIC stimulation preceded by either DMSO or JQ1 treatment in four melanoma cell lines. *n* ≥ 3 biological replicates/cell line. **L,** Representative histogram plot for A375 from (**G**). **M,** Cell surface expression of HLA-I assessed by flow cytometry after 48 hours of pIC stimulation preceded by either DMSO or JQ1 treatment in the colorectal cancer cell line COLO320. *n* = 3 biological replicates. **N,** Cell surface expression of HLA-I in A375 after JQ1/pIC in the presence or absence of an IFNα/βR1-blocking antibody (2 µg/mL). *n* = 3 biological replicates. **O,** HLA-I expression in ANRU cells upon concomitant treatment with JQ1 and/or nontargeting control siRNA (NTC) or siRNA targeting TLR3, RIG-I, or MDA-5, prior to transfection with pIC. *n* = 3 biological replicates. Error bars represent mean ± standard deviation. Statistical analysis was performed using paired one- or two-way ANOVA with Holm–Šidák correction for multiple comparisons. *P* values <0.05 are indicated above the relevant comparisons. geoMFI, geometric mean fluorescence intensity. [**A,** Created in BioRender. Wickstrom, S. (2026) https://BioRender.com/npulau2.]

BET proteins are known to regulate T-cell immunity, yet their role in regulating dsRNA-sensing pathways in cancer is unclear (7). To address this, melanoma cells were pretreated for 72 hours with the BETi JQ1 and subsequently transfected with the viral dsRNA mimic pIC to promote antiviral signaling through engagement of RLRs (Fig. 1A; ref. 13). We observed that pIC induced phosphorylation of TBK1 and IRF3, which was markedly enhanced if tumor cells were pretreated with JQ1 (Fig. 1B–D). Consistent with these findings, this combined treatment with JQ1 and pIC (JQ1/pIC) amplified gene expression of the RLRs *RIG-I* and *MDA-5*, as well as that of proinflammatory cytokines (*IFNβ*, *CXCL10*, and *CXCL11*; Fig. 1E–H). The ability of JQ1 to enhance pIC-induced secretion of cytokines, including type I/III IFNs and CXCL10, was confirmed by ELISA (Fig. 1I and J). Strikingly, treatment of tumor cells with JQ1 alone had a minimal effect on gene and protein expression levels of these same targets. Along with the enhanced downstream signaling following RLR activation, combined JQ1/pIC treatment significantly upregulated HLA-I cell surface expression, whereas JQ1 alone did not (Fig. 1K and L). This effect was independent of melanoma differentiation status, as both differentiated (A375) and undifferentiated (KADA) cell lines responded similarly (Supplementary Fig. S2). Although higher JQ1 doses were required for the vemurafenib-resistant A375 subclone A375_VR4_, which is characterized by a strongly dedifferentiated phenotype, JQ1 still enhanced pIC-induced HLA-I upregulation in a dose-dependent manner (Supplementary Fig. S3A).

To confirm whether this effect could be achieved with other BETi, ANRU and A375 cells were pretreated in the same way as JQ1 with the BETi I-BET151 or dBET1 before being transfected with pIC. Although I-BET151 indeed enhanced HLA-I surface induction when combined with pIC, dBET1 did not (Supplementary Fig. S4A and S4B). Strikingly, although all evaluated BETi reduced BRD4 protein levels, only dBET1 induced a concomitant decrease in the expression of BRD2 (Supplementary Fig. S4C–S4F). These data indicate that BETi with a mechanism of action similar to that of JQ1, such as I-BET151, may be more effective at sensitizing tumor cells to dsRNA-sensing pathways than BET degraders like dBET1.

Targeting BET proteins and activating RIG-I signaling have been shown to enhance tumor immunogenicity in multiple types of cancer, including colorectal cancer (14, 15). Indeed, applying our JQ1/pIC treatment approach to the colorectal cancer cell line COLO320 also led to the marked enhancement of HLA-I induction (Fig. 1M), suggesting a broader relevance of our approach beyond melanoma.

To investigate what molecular mechanisms underlie the enhanced HLA-I induction observed upon combined JQ1/pIC treatment, we next examined the role of type I IFN signaling and cytosolic dsRNA receptors. Blocking IFNα/βR1 signaling in A375 cells partially reduced JQ1/pIC-induced HLA-I expression, demonstrating that enhanced type I IFN production contributes to, but does not fully account for, increased HLA-I levels (Fig. 1N). However, silencing of RIG-I or MDA-5 attenuated the enhancement of HLA-I induction observed after combined JQ1/pIC treatment. Importantly, no significant effect on HLA-I induction was seen after TLR3 silencing (Fig. 1O). These results indicate that the potentiating effect of JQ1 on pIC-induced HLA-I expression is primarily dependent on cytosolic RLRs rather than on TLR3 signaling. Overall, our findings show that prolonged BET inhibition sensitizes tumor cells to stimulation by RLR agonists, which leads to amplified antiviral signaling, increased production of IFN and other cytokines, and antigen presentation across tumor types and differentiation states.

### Transcriptional analysis reveals an enhanced proinflammatory and antiviral state upon combination of JQ1 and pIC

**Figure 2.**
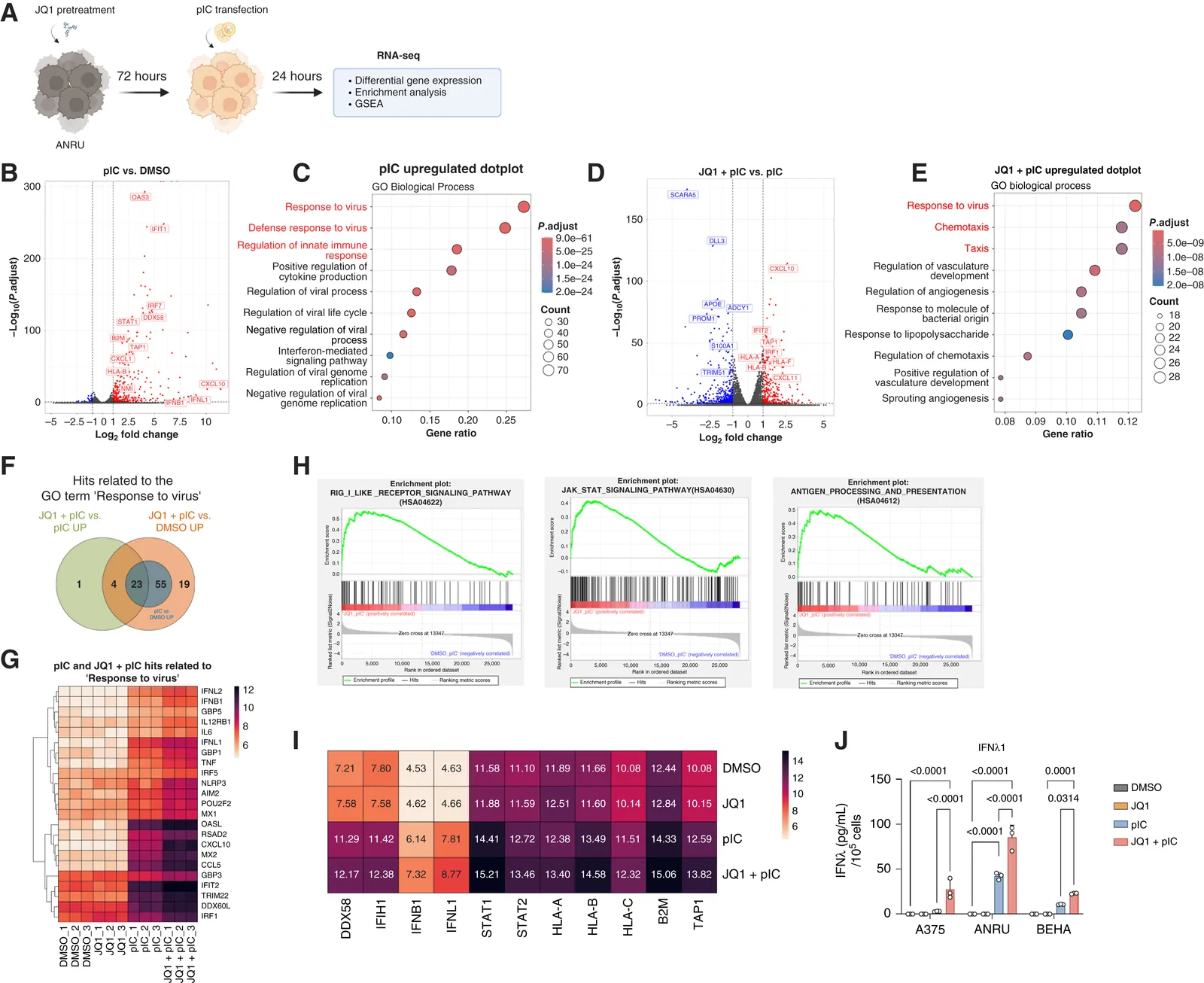
Transcriptional analysis reveals an enhanced proinflammatory and antiviral state upon combined JQ1/pIC treatment. **A,** Experimental setup for RNA-seq experiment. **B,** Volcano plot of differentially expressed genes (DEG) between pIC- and DMSO-treated ANRU tumor cells. **C,** Dot plot of the top 10 enriched GOs in pIC versus DMSO. **D,** Volcano plot of DEGs between JQ1/pIC- and pIC-treated ANRU tumor cells. **E,** Dot plot of the top 10 enriched GOs in JQ1/pIC versus pIC. **F,** Venn diagrams displaying the number of significantly upregulated genes between various treatment groups from the GO term “Response to virus.” **G,** Heatmap displaying the expression of genes in DMSO-, JQ1-, pIC-, and JQ1/pIC-treated cells found differentially expressed between pIC versus DMSO and JQ1/pIC versus pIC in GO terms “Response to virus” (23 genes). **H,** Kyoto Encyclopedia of Genes and Genomes (KEGG) enrichment plots of pathways enriched in JQ1/pIC versus pIC. **I,** Heatmap of selected genes from KEGG pathways displayed in (**H**). **J,** IFNλ1 concentration in supernatants from treated A375, ANRU, and BEHA cells. *n* = 3 biological replicates/cell line. Error bars represent mean ± standard deviation. Statistical analysis was performed using paired one- or two-way ANOVA with Holm–Šidák correction for multiple comparisons. *P* values <0.05 are indicated above the relevant comparisons. geoMFI, geometric mean fluorescence intensity. [**A,** Created in BioRender. Wickstrom, S. (2026) https://BioRender.com/b31gu8z.]

To further characterize the phenotype of BETi and pIC-treated tumor cells, bulk RNA-seq was performed (Fig. 2A). Cells treated with pIC displayed a marked increase in the expression of dsRNA-sensing molecules (*OAS3*, *IFIT1*, *DDX58*, and *IRF7*), IFN-induced molecules involved in antigen processing and presentation (*STAT1*, *TAP1*, *B2M*, *HLA-A*, and *HLA-B*), and proinflammatory cytokines/chemokines (*IFNB1*, *IFNL1*, *CXCL10*, and *CXCL11*; Fig. 2B). In line with this, dsRNA stimulation led to activation of cellular programs involved in “Response to virus” and “Regulation of innate immune response” (Fig. 2C; Supplementary Fig. S5A).

Comparison of JQ1/pIC- and pIC-treated cells revealed enhanced expression of chemokines (*CXCL10* and *CXCL11*) and HLA molecules (*HLA-A*, *HLA-B*, and *HLA-F*; Fig. 2D), consistent with the protein-level changes described in Fig. 1. Compared with pIC treatment alone, the combination treatment also resulted in a set of downregulated genes, reflecting the transcriptional effects of JQ1 and consistent with prolonged JQ1 exposure alone (Fig. 2D; Supplementary Fig. S5B). Enrichment analysis of genes upregulated by JQ1/pIC versus pIC treatment confirmed that boosting of antiviral signaling by JQ1 is associated with an enrichment of genes involved in “Defense response to virus” and “Chemotaxis” programs (Fig. 2E; Supplementary Fig. S5C).

Consistent with our previous observations, we found that JQ1 essentially primes tumor cells for the enhancement of antiviral signaling upon stimulation with an RLR agonist, as the induction of proinflammatory cytokines induced by pIC was potentiated in JQ1-pretreated cells, whereas treatment with JQ1 alone had virtually no effect on these same pathways (Fig. 2F and G; Supplementary Fig. S5D and S5E). Additionally, GSEA confirmed the enhanced immunogenicity of JQ1/pIC-treated cells, as they were transcriptionally enriched in genes related to RIG-I signaling, JAK–STAT signaling, and antigen processing and presentation (Fig. 2H and I). Notably, JQ1/pIC-treated tumor cells displayed enhanced gene expression and production of type III IFNs (Fig. 2I and J).

Together, these findings demonstrate that BET inhibition broadly reshapes the transcriptional response to dsRNA stimulation, reinforcing antiviral signaling and immunostimulatory gene programs.

### Prolonged BET inhibition rewires antiviral pathways and eliminates negative regulators of dsRNA-sensing pathways

**Figure 3.**
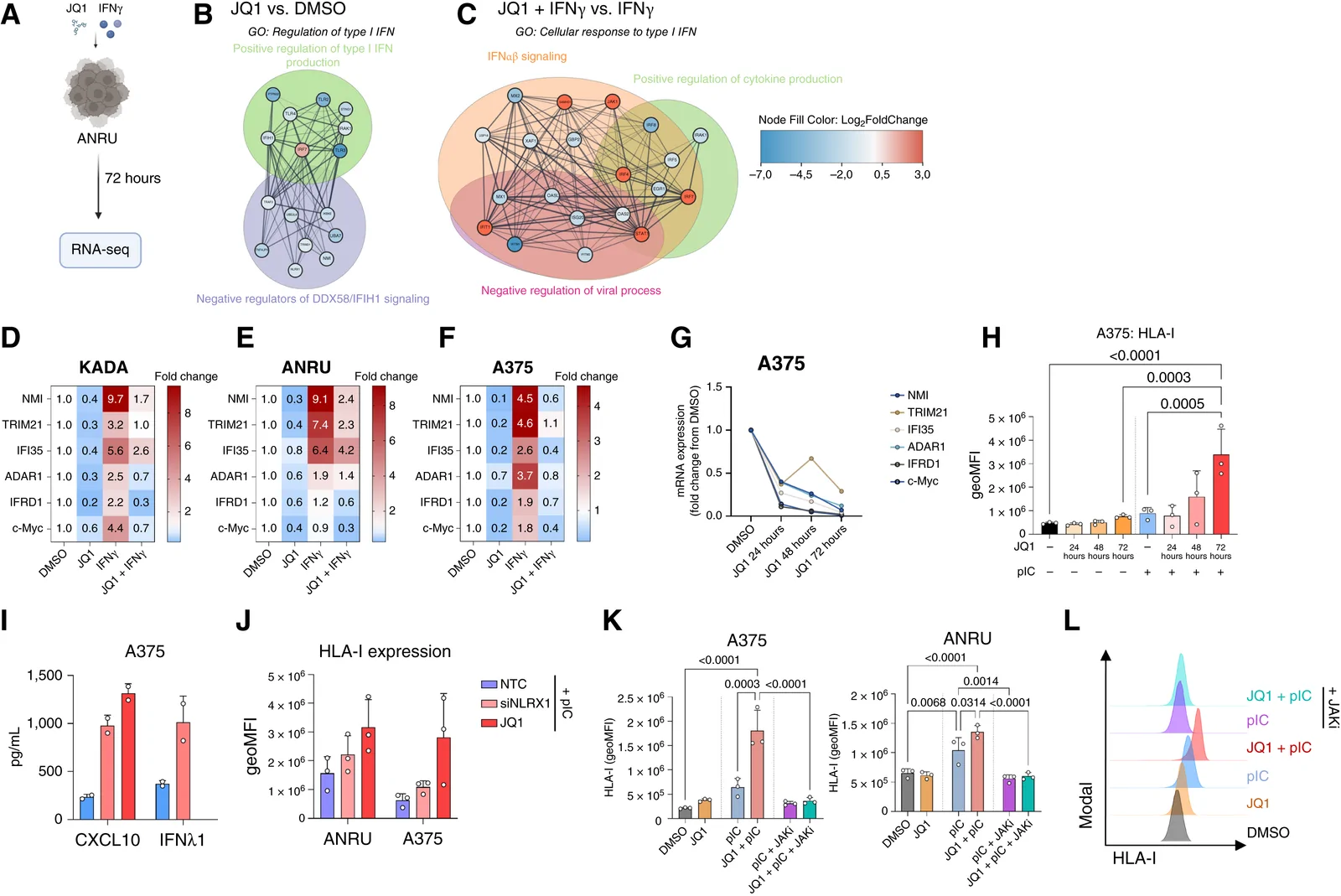
Prolonged BET inhibition rewires antiviral pathways and depletes tumors of negative regulators of dsRNA-sensing pathways. **A,** Schematic overview of the RNA-seq experiment related to (**B**) and (**C**). **B,** Cytoscape network of significantly regulated genes by JQ1 compared with DMSO related to the GO term “Regulation of type I interferon.” Node color represents upregulated (red) or downregulated (blue) genes in JQ1 versus DMSO. **C,** Network of significantly regulated genes by JQ1 + IFNγ compared with IFNγ-treated cells related to the GO term “Cellular response to type I interferon.” Node color represents upregulated (red) or downregulated (blue) genes in JQ1 + IFNγ versus IFNγ. **D,** Heatmap of selected genes from qPCR after 48 hours of JQ1-, IFNγ- or JQ1 + IFNγ-treated KADA, (**E**) ANRU, and (**F**) A375. Data are represented as mean fold change from DMSO. *n* = 2 biological replicates/cell line. **G,** Gene expression of negative regulators of antiviral signaling after 24, 48, and 72 hours of JQ1 treatment in A375. Mean of *n* = 3 technical replicates. **H,** Cell surface expression of HLA-I was assessed by flow cytometry after A375 cells were subjected to pretreatment with JQ1 alone for either 24, 48, or 72 hours and/or stimulation with pIC for 48 hours. *n* = 3 biological replicates. **I,** CXCL10 and IFNλ1 concentrations were measured by ELISA after pIC transfection of cells pretreated with nontargeting control siRNA (NTC), NLRX1-targeting siRNAs (siNLRX1), or JQ1. *n* = 2 biological replicates. **J,** HLA-I expression in A375 and ANRU cells after NTC, siNLRX1, or JQ1 pretreatment and pIC transfection. *n* = 3 biological replicates. **K,** HLA-I expression of A375 and ANRU upon JQ1 and/or pIC in the presence or absence of a JAK1/2 inhibitor (ruxolitinib, 2 µmol/L). **L,** Representative flow cytometry histogram from A375-treated cells in (**K**). Error bars represent mean ± standard deviation. Statistical analysis was performed using paired one- or two-way ANOVA with Holm–Šidák correction for multiple comparisons. *P* values <0.05 are indicated above the relevant comparisons. geoMFI, geometric mean fluorescence intensity. [**A,** Created in BioRender. Wickstrom, S. (2026) https://BioRender.com/xoxbyk3.]

We confirmed that BETi treatment sensitizes tumor cells to dsRNA sensing. As these findings seem to contrast with reports of BETi-mediated suppression of proinflammatory pathways (16), we sought to understand how JQ1 amplifies dsRNA-sensing pathways. For this, transcriptomic analysis of tumor cells treated with JQ1 and/or IFNγ was performed (Fig. 3A).

Given the broad and pleiotropic effects of BET inhibition and IFNγ treatment on gene expression, we focused on GO terms involved in antiviral processes, specifically “Regulation of type I interferon” and “Cellular response to type I interferon.” Of note, enrichment analysis of JQ1-treated cells showed repressed cellular programs involved in cell adhesion, extracellular matrix (ECM) organization, wound healing, and angiogenesis—programs found upregulated in tumors that fail to respond to anti–PD-1 therapy (Supplementary Fig. S6A and S6B; ref. 17).

JQ1-treated cells displayed decreased expression of a proportion of IFN-stimulated genes (ISG) involved in the positive regulation of RLR signaling, notably *OAS*, *TLR3*, and *MDA-5* (Fig. 3B and C). However, JQ1 treatment also led to downregulation of known negative regulators of antiviral signaling, including *NMI*, *TRIM21*, *NLRX1*, and *TNFAIP3* (18, 19). When comparing JQ1 + IFNγ versus IFNγ datasets, JQ1 treatment was found to enhance a subset of ISGs involved in the positive regulation of JAK–STAT signaling (*JAK1*, *STAT1*, and *IRF7*), as well as suppress IFN-induced genes involved in antiviral processes (*OASL*, *OAS2*, *IRF8*, and *IRF5*; Fig. 3C).

Transcriptional repression of *NMI*, *TRIM21*, and *IFI35* by JQ1 in KADA, ANRU, and A375, as well as three other negative regulators of cellular responses to viral sensing pathways (*ADAR1*, *IFRD1*, and *c-MYC*), was confirmed by qPCR (Fig. 3D–F). Importantly, exposure to JQ1 treatment led to a time-dependent downregulation of these genes in A375 cells (Fig. 3G), which coincided with a time-dependent enhancement of HLA-I upon combined JQ1/pIC treatment (Fig. 3H).

*MYC* downregulation by JQ1 was expected given that BETi were originally designed for MYC targeting (5). Given the role of MYC in dampening antiviral responses in cancer (20), we speculated that the boosting of antiviral signaling by JQ1 might be mediated by MYC repression. However, only 5 of 230 *MYC* targets, including *MYC* itself, were downregulated by JQ1 in ANRU (Supplementary Fig. S7A). Using pharmacologic approaches, we could not reproducibly phenocopy the effect of JQ1 in A375 and ANRU cells by pretreating tumors with the MYC inhibitors MYCMI-6 or MYC-RIBOTAC (Supplementary Fig. S7B–S7D). Although MYC downregulation may contribute to BETi-induced sensitivity to pIC, these data suggest that BET proteins control MYC-independent mechanisms regulating RLR signaling pathways.

ADAR1 and NLRX1 have been shown to dampen antiviral signaling by posttranscriptionally repressing MAVS, a mitochondrial protein and essential hub for signal transduction once cytosolic RNA is detected in a cell (21). Exposure of ANRU and A375 to JQ1 enhanced MAVS protein expression, even upon slight yet consistent downregulation by IFNγ, and the expression of MAVS was found to be negatively correlated with that of *ADAR1* in our cells (Supplementary Fig. S8A and S8B). Additionally, suppression of ADAR or NLRX1 in A375 cells using silencing RNAs led to a modest increase in MAVS expression (Supplementary Fig. S8C), and NLRX1-suppressed cells exhibited enhanced sensitivity to pIC, as indicated by increased CXCL10/IFNλ1 production and HLA-I expression, albeit to a lesser extent than JQ1-pretreated cells (Fig. 3I and J). This implies that among other means, JQ1 can sensitize tumor cells to RLR signaling by alleviating posttranscriptional mechanisms of MAVS suppression.

Given that MAVS signaling can cross-talk with several other PRR signaling pathways and that NLRX1 has been described to also suppress TLR-induced NF-κB signaling, we wondered whether JQ1 treatment might sensitize tumor cells to other non-RLR agonists (21). To assess this, JQ1-pretreated A375 cells were subjected to a plethora of TLR and PRR agonists, and HLA-I levels were measured. Surprisingly, only transfection with pIC led to enhanced HLA-I expression, which was further induced by JQ1 pretreatment (Supplementary Fig. S9A). These data indicate that BETi selectively sensitizes tumor cells to the immunogenicity-enhancing effects of viral dsRNA mimics delivered to the cytosol. Finally, knowing that activation of RIG-I/MDA-5 signaling in tumors can promote MHC class I expression in a JAK-independent manner (22), we investigated whether JQ1 and pIC might also do so. HLA-I upregulation by pIC alone or by combined JQ1/pIC treatment was completely abrogated in the presence of a JAK1/2 inhibitor (ruxolitinib; Fig. 3K and L), which demonstrates that the induction of HLA-I by pIC alone or combined JQ1/pIC treatment requires intact JAK–STAT signaling in our model.

Overall, our findings indicate that JQ1 potentiates dsRNA signaling by suppressing multiple inhibitory regulators, including MAVS repressors such as NLRX1 and ADAR1, thereby derepressing the activation of RLR-sensing pathways to promote cytokine production and JAK-dependent antigen presentation.

### Enhanced antiviral signaling in melanoma promotes the activation of cytolytic autologous TILs in an antigen-dependent manner

Next, we wondered whether the sensitizing effect of JQ1 on dsRNA signaling would have any functional effect on tumor recognition by T cells, as activation of the RIG-I/MDA-5 pathway promotes CD8^+^ T cell–mediated antitumor immunity (23). For this, tumor cells were treated and cocultured with autologous TILs, and recognition was measured by IFNγ ELISA and flow cytometry.

**Figure 4.**
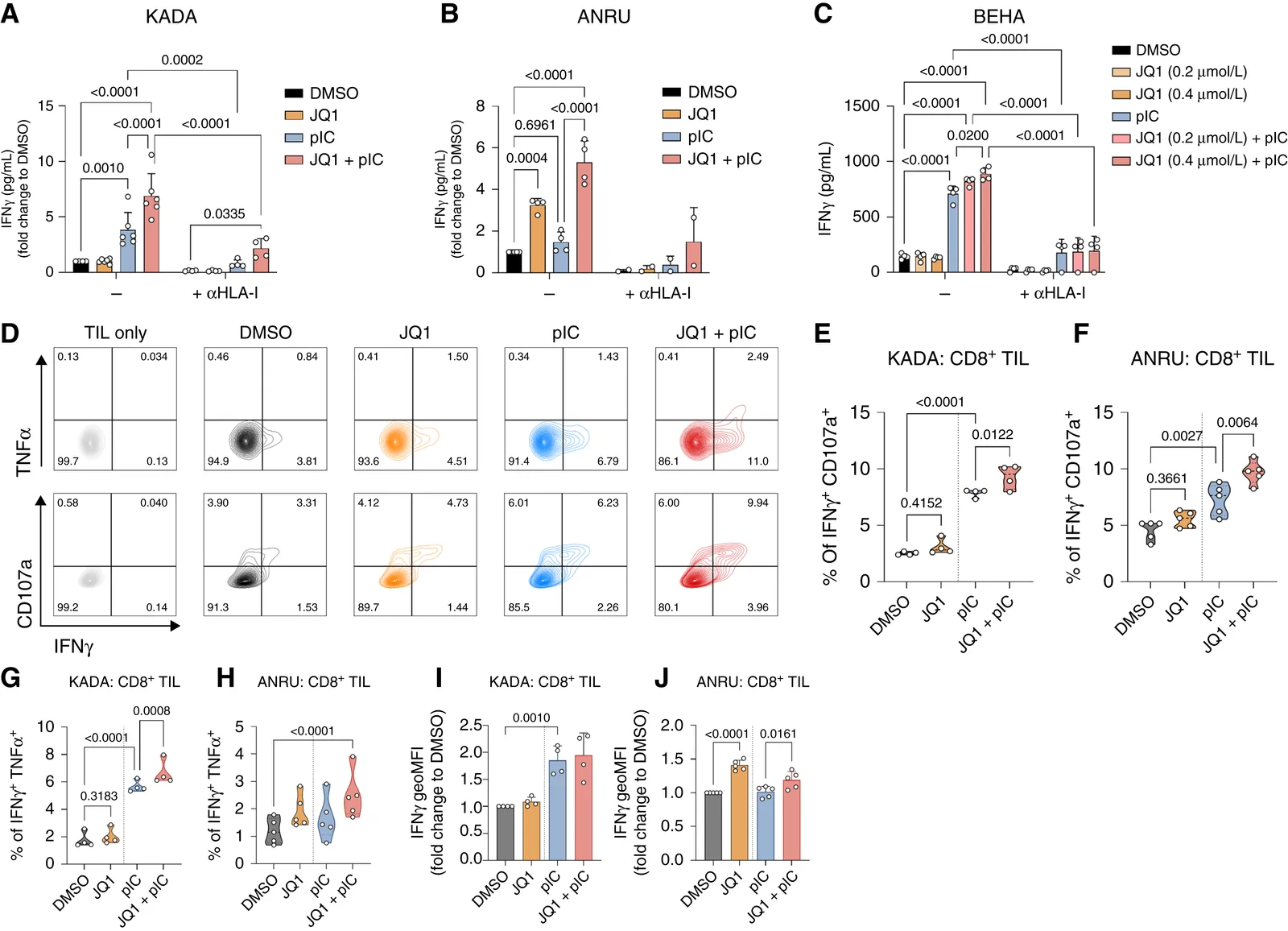
Tumor cells treated with JQ1 promote activation of cytotoxic TILs in melanoma when combined with dsRNA agonist. **A,** Bar graph displaying IFNγ secretion measured by ELISA after coculture of autologous TILs with KADA (*n* = 6 biological replicates), (**B**) ANRU (*n* = 4 biological replicates), and (**C**) BEHA (*n* = 4 biological replicates) tumor cells after treatment with DMSO, JQ1, pIC, or JQ1/pIC, in the presence or absence of an HLA-I–blocking antibody. **D,** Representative flow cytometry plots from coculture of TILs with treated ANRU tumor cells gated on live CD3^+^CD8^+^ and IFNγ^+^CD107a^+^ (bottom) or IFNγ^+^TNFα^+^ (top) T cells. **E,** Violin plots display the frequency of IFNγ^+^CD107a^+^ in CD8^+^ T cells in KADA and (**F**) ANRU TILs. **G,** Violin plots display the frequency of IFNγ^+^TNFα^+^ in CD8^+^ T cells in KADA and (**H**) ANRU TIL. **I,** Mean IFNγ expression in live CD3^+^CD8^+^IFNγ^+^ KADA and (**J**) ANRU TILs. Error bars represent mean ± standard deviation. Statistical analysis was performed using paired one- or two-way ANOVA with Holm–Šidák correction for multiple comparisons. *P* values <0.05 are indicated above the relevant comparisons. geoMFI, geometric mean fluorescence intensity.

TILs cocultured with pIC-treated KADA or BEHA cells, but not with JQ1-treated tumor cells, strongly responded by secreting IFNγ. With ANRU cells, this observation was reversed (Fig. 4A–C). However, for all three tumor lines, combined JQ1/pIC treatment resulted in the strongest tumor recognition by autologous TILs. In BEHA, pIC-induced IFNγ production by TILs was enhanced by JQ1 in a dose-dependent manner, even in the absence of an effect on T-cell activation from JQ1-only treated tumors (Fig. 4C). In all patients, tumor recognition was HLA-I/peptide-dependent, as blockade of HLA-I on tumor cells prior to the addition of autologous TILs to the coculture incapacitated their IFNγ production. In line with this, JQ1/pIC-treated tumor cells induced a higher frequency of activated CD107a^+^/IFNγ^+^ CD8^+^ TILs as opposed to single-agent treatments (Fig. 4D–F). ANRU and KADA CD8^+^ TILs also displayed a capacity for TNFα production, which was significantly enhanced following combination treatment of the autologous tumor cells (Fig. 4G and H).

Of note, CD4^+^ TILs were largely unreactive to treated KADA tumors upon short-term coculture, and a small fraction of CD4^+^IFNγ^+^ T cells in ANRU were found to be suppressed by JQ1 (Supplementary Fig. S10A–S10G). This coincided with tumoral HLA-II expression, which was downregulated by JQ1 alone in ANRU and, to a lesser extent, A375 cells (Supplementary Fig. S10H).

Lastly, quantification of mean IFNγ expression in CD8^+^IFNγ^+^ cells showed increased IFNγ production by pIC in KADA and JQ1 in ANRU (Fig. 4I and J), consistent with our ELISA results.

Overall, BET inhibition combined with dsRNA agonists enhances HLA-I–dependent activation of CD8^+^ autologous TILs.

### Enhanced activation of neoantigen-specific and tumor-associated antigen–specific CD8^+^ TILs upon JQ1 and/or pIC treatment of cancer cells

**Figure 5.**
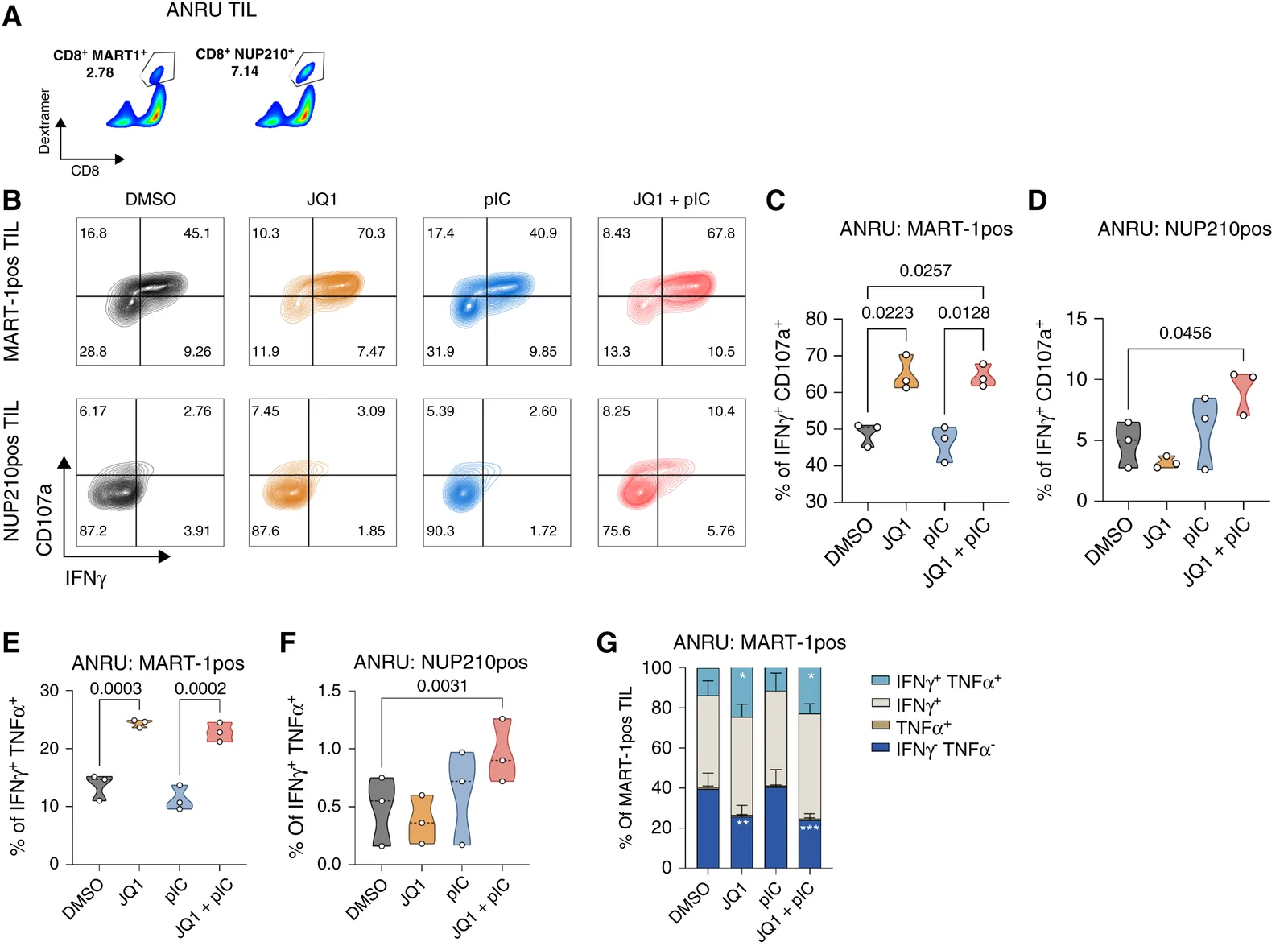
Enhanced activation of neoantigen/TAA-specific CD8^+^ TILs using JQ1 and/or pIC treatment in cancer cells. **A,** Proportion of CD8^+^ MART-1pos (MART-1^+^) T cells and CD8^+^ NUP210pos (NUP210^+^) T cells in CD3^+^ ANRU TIL. **B,** Representative flow cytometry plots for IFNγ^+^CD107a^+^ T cells gated on MART-1pos TIL and NUP210pos TIL after coculture with treated ANRU tumor cells. **C,** Frequency of IFNγ^+^CD107a^+^ MART1pos and (**D**) IFNγ^+^CD107a^+^ NUP210pos T cells after coculture with treated ANRU tumor cells. *n* = 3 biological replicates. **E,** Frequency of IFNγ^+^TNFα^+^ for MART-1pos T cells and (**F**) IFNγ^+^TNFα^+^ NUP210pos T cells. *n* = 3 biological replicates. **G,** Proportion of IFNγ^+^, TNFα^+^, IFNγ^−^TNFα^−^, and IFNγ^+^TNFα^+^ MART-1pos TILs after coculture with treated ANRU tumor cells. *n* = 3 biological replicates. Error bars represent mean ± standard deviation. Statistical analysis was performed using paired one- or two-way ANOVA with Holm–Šidák correction for multiple comparisons. *P* values <0.05 are indicated above the relevant comparisons.

To be effective, antitumoral T-cell responses rely on adequate presentation of HLA-bound peptides derived from tumor-associated antigens (TAA) and neoantigens (24). We next investigated how our treatment might affect the activation of antigen-specific T cells. To this end, ANRU TILs specific for HLA-A*02:01–bound peptides were stained using dextramers. Previous work in our lab revealed that ANRU TILs contain CD8^+^ T cells specific for peptides derived from the MART-1 protein and the mutated NUP210 protein (NUP210^mut^; ref. 11). In keeping with this, ANRU CD8^+^ TILs contained MART-1–specific (MART-1pos) and NUP210^mut^-specific T-cell populations (NUP210pos; Fig. 5A). Upon coculture, tumor cells treated with JQ1 alone or JQ1/pIC, but not pIC alone, increased the proportion of activated IFNγ^+^CD107a^+^ and IFNγ^+^TNFα^+^ MART-1pos T cells (Fig. 5B–G). These data support claims that RLR signaling in melanoma can promote dedifferentiation and loss of melanocytic antigens (25). Indeed, pIC downregulated the expression of MART-1 and gp100 while upregulating NGFR levels, whereas JQ1 pretreatment increased MART-1 expression (Supplementary Fig. S11). JQ1 also enhanced NGFR expression and further suppressed gp100 expression in ANRU, suggesting that JQ1 can augment aspects of pIC-induced dedifferentiation while promoting MART-1 expression.

In regard to neoantigen-specific T-cell responses, only combined JQ1/pIC treatment significantly increased the frequency of activated NUP210pos TILs (Fig. 5B, D, and F), consistent with increased HLA-I expression (related to Fig. 1K).

Overall, combined BETi and pIC treatment enhances tumoral immunogenicity and activation of both TAA- and neoantigen-specific T cells.

### Enhanced dsRNA signaling through BETi promotes ICD and DC maturation

Antiviral signaling promotes ICD in tumor cells, an important mechanism for the activation and recruitment of immune cells, particularly antitumoral cross-presenting conventional type I DCs (26–28). We therefore assessed whether BETi enhances RLR-induced ICD.

**Figure 6.**
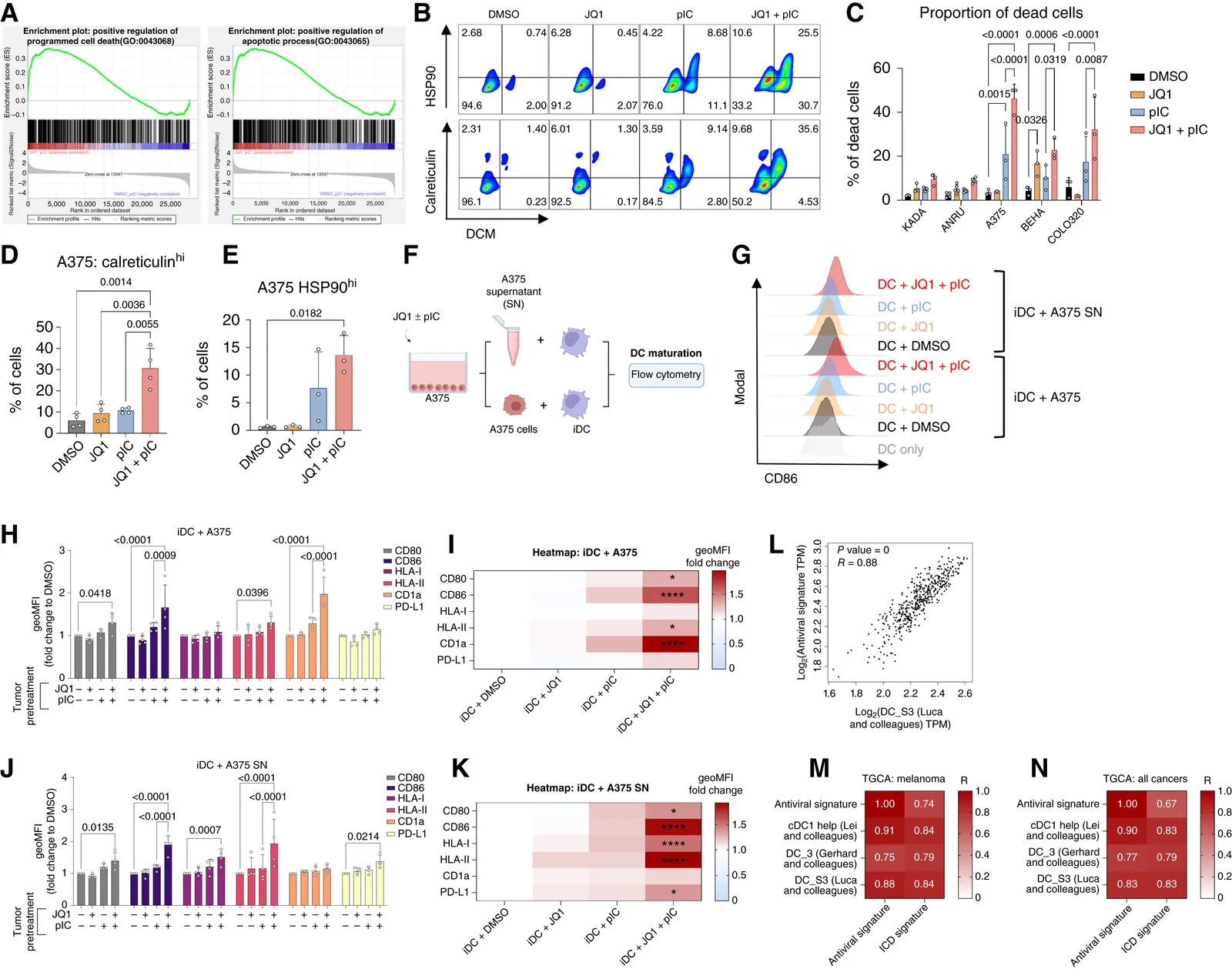
Enhanced dsRNA signaling through BETi promotes ICD and DC maturation. **A,** GO enrichment plots from JQ1/pIC versus pIC comparison in ANRU related to the GO terms “Positive regulation of programmed cell death” and “Positive regulation of apoptotic process.” **B,** Representative flow cytometry gating for dead cells, calreticulin, and HSP90 in treated A375 cells. **C,** Proportion of dead cells in KADA, ANRU, A375, BEHA, and COLO320 cells after JQ1 and/or pIC treatment. *n* ≥ 3 biological replicates/cell line. **D,** Bar graphs indicating the frequency of calreticulin^hi^ (*n* = 4 biological replicates) and (**E**) HSP90^hi^ (*n* = 3 biological replicates) A375 cells after JQ1 and/or pIC treatment. **F,** Experimental setup for DC experiments. **G,** Representative histogram plots of CD86 in DCs after exposure to treated A375 cells or A375 SN. **H,** Expression of CD80, CD86, HLA-I, HLA-II, CD1a, and PD-L1 displayed as bar graphs or (**I**) heatmap after exposure of iDCs to treated A375 cells for 48 hours. Data are presented as fold change to iDCs exposed to DMSO-treated A375 cells. *n* = 4 biological replicates. **J,** Expression of CD80, CD86, HLA-I, HLA-II, CD1a, and PD-L1 displayed as bar graphs or (**K**) heatmap after exposure of iDCs to treated A375 SN for 48 hours. Data are presented as fold change to iDCs exposed to control A375 SN. *n* = 4 biological replicates. **L,** Correlation plot between the expression of the DC_S3 signature (Luca and colleagues) and the antiviral signature in TCGA patients with melanoma. **M,** Correlation analysis of antiviral signature, ICD signature, and 3 single-cell RNA-seq DC signatures in the TCGA cohort of patients with melanoma and (**N**) in all patients with cancer from TCGA. Error bars represent mean ± standard deviation. Statistical analysis was performed using paired one- or two-way ANOVA with Holm–Šidák correction for multiple comparisons. *P* values <0.05 are indicated above the relevant comparisons. geoMFI, geometric mean fluorescence intensity. [**F,** Created in BioRender. Wickstrom, S. (2026) https://BioRender.com/dbfr9mh.]

RNA-seq analysis of ANRU revealed an enrichment of proapoptotic pathways following treatment with JQ1 and pIC (Fig. 6A). To address this *in vitro*, tumor cell viability and surface expression of ICD markers calreticulin and HSP90 were quantified (29, 30). Cells subjected to combined JQ1/pIC treatment exhibited the highest levels of cell death compared with either single agent (Fig. 6B and C). Moreover, JQ1 enhanced pIC-induced surface expression of calreticulin and HSP90 in A375 cells (Fig. 6B, D, and E), and a similar upregulation of calreticulin was observed in BRAFi-resistant A375_VR4_ cells (Supplementary Fig. S12A). This indicates that BET inhibition augments pIC-induced ICD, which was functionally validated by coculturing treated A375 cells (A375) or their supernatants (A375 SN) with iDCs for 48 hours, followed by analysis of maturation markers (Fig. 6F). Both pIC-treated and, more prominently, JQ1/pIC-treated A375 cells or their supernatants enhanced the expression of CD80, CD86, HLA-I, HLA-II, CD1a, and PD-L1 on DCs (Fig. 6G–K). Next, we decided to further validate these findings using the TCGA melanoma database. Indeed, several studies have indicated that expression of gene signatures of DC subsets is associated with improved responses to immunotherapy and clinical outcomes in patients with cancer (31–33). Using our transcriptomic data, we computed an antiviral signature using the top 100 upregulated genes in pIC-treated ANRU cells, ranked on adjusted *P* values. A strong positive correlation was found between these described DC signatures, our antiviral signature, and an ICD signature, both in patients with melanoma and in a pan-cancer analysis from TCGA (Fig. 6L–N). Thus, our data suggest that BETi can amplify ICD in cancer cells upon activation of dsRNA-sensing pathways, which is associated with the maturation and activation of DC subsets that are implicated in improved responses to immunotherapy.

### Antiviral signaling in tumor cells is associated with improved immunotherapy efficacy and survival of patients with cancer

Next, we explored whether our *in vitro* findings might have clinical implications for patients with cancer. Jerby-Arnon and colleagues generated immune exclusion and immune resistance signatures using single-cell RNA-seq and bulk RNA-seq of melanoma biopsies (34). These signatures consist of transcripts positively (up) or negatively (down) associated with immune exclusion and resistance.

**Figure 7.**
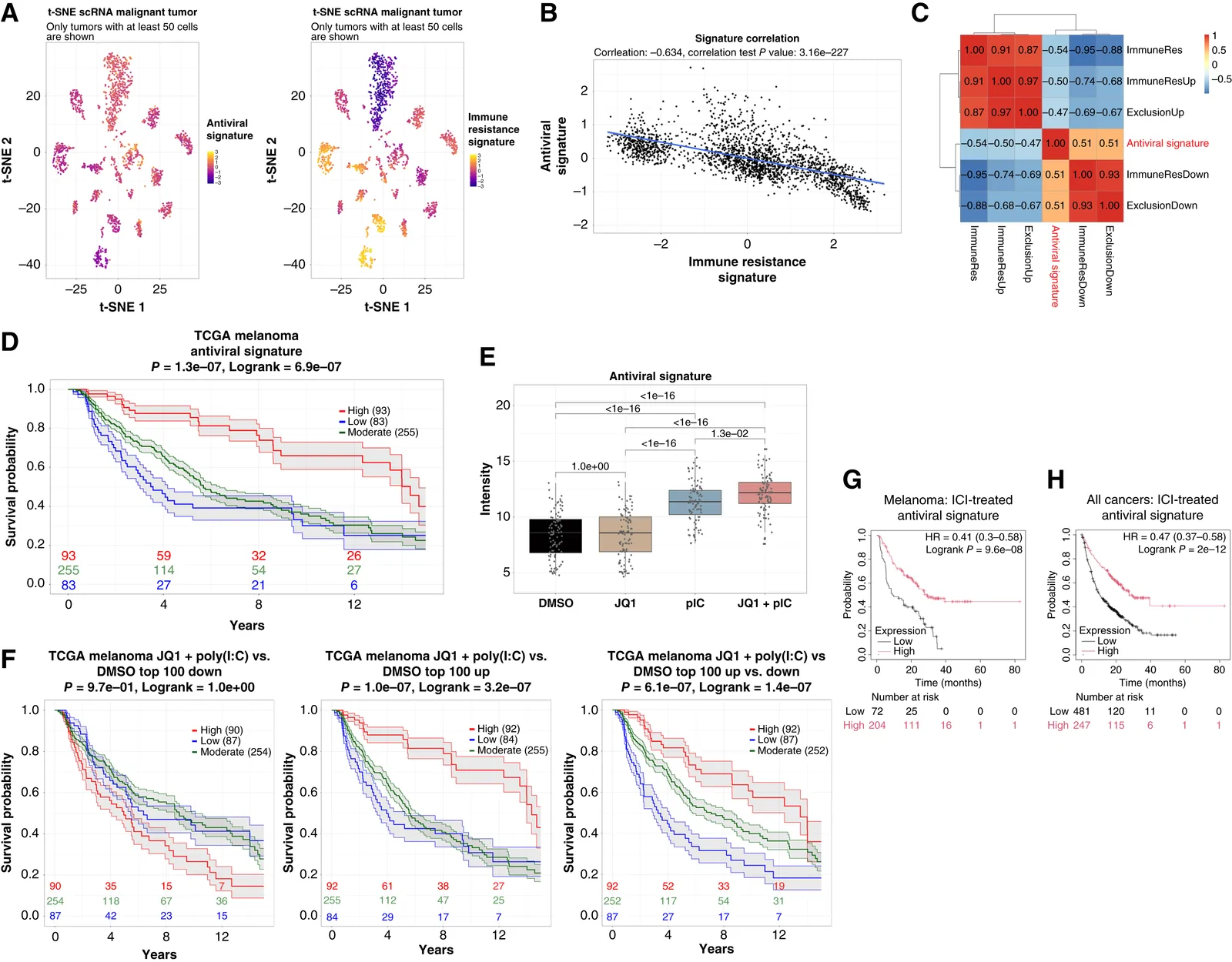
Antiviral signaling correlates with improved immunotherapy efficacy and survival of patients with cancer. **A,** t-distributed stochastic neighbor embedding (t-SNE) plot displaying the inverse correlation between the expression of the antiviral signature and the immune resistance signature in melanoma cells, quantified in (**B**). **C,** Heatmap showing the level of correlation between single-cell RNA-seq signatures of immune resistance/exclusion and our antiviral signature in TCGA patients with melanoma. **D,** Expression of the antiviral signature predicts survival of patients with melanoma in the TCGA cohort. Kaplan–Meier (KM) curves are stratified by high (top 20%), low (bottom 20%), or moderate (remainder) expression of the antiviral signature. The number of subjects at risk is indicated at the bottom of the KM curves for four time points. P, COX regression *P* value. **E,** Expression of the antiviral signature is enhanced in ANRU upon JQ1 pretreatment. **F,** Survival analysis using JQ1/pIC downregulated, upregulated, or up–down gene signatures in TCGA patients with melanoma. **G,** Expression of the antiviral signature correlates with improved OS in immunotherapy-treated melanoma and (**H**) pan-cancer patients. KM curves are stratified by high (top 20%) and low (bottom 20%) expression of the antiviral signature.

Using Jerby-Arnon’s single-cell cohort, the expression of our antiviral signature negatively correlated with the immune resistance signature in melanoma cells (*r* = −0.634; Fig. 7A and B). This was confirmed in the TCGA melanoma cohort, in which the antiviral signature negatively correlated with immune resistance/exclusion up genes and positively correlated with genes found downregulated in immune resistant/excluded tumors (Fig. 7C).

In line with this, the expression of antiviral genes was positively correlated with immune cell infiltration, in addition to the previously observed correlation with DC gene signatures (Supplementary Fig. S13A). Additionally, high expression of antiviral genes was associated with improved OS in this cohort (*P* = 1.2 × 10^−7^; Fig. 7D).

As previously outlined, the majority of the top 100 genes found to be upregulated in JQ1/pIC overlapped with those induced by pIC alone (81/100; Supplementary Fig. S13B), whereas genes downregulated in combination-treated cells were attributable to JQ1 treatment. JQ1 treatment increased the expression of this antiviral signature in ANRU tumor cells when combined with pIC (Fig. 7E). Similarly, a gene signature derived from JQ1/pIC-treated cells strongly predicted patient survival in this cohort (Fig. 7F). Notably, the top 100 downregulated genes in JQ1/pIC-treated cells were involved in ECM production and cholesterol metabolism (Supplementary Fig. S13C). Low expression of these genes further improved discrimination and prediction of OS when combined with an antiviral signature (Fig. 7F), which points to the role of clinically relevant, antiviral-independent programs suppressed by JQ1.

Finally, in patients who underwent immunotherapy, the expression of pIC-induced genes predicted OS in both melanoma and pan-cancer cohorts (Fig. 7G and H).

Overall, these data highlight the clinical relevance of tumor-intrinsic antiviral programs associated with immune infiltration, immunotherapy response, and improved survival of patients with cancer.

## Discussion

This study elucidates the role of BET proteins in regulating RLR signaling within tumor cells and reveals that prolonged tumor cell exposure to BETi broadly modulates key regulators of innate antiviral immunity, which potently sensitizes them to agonists of the cytosolic dsRNA receptors RIG-I and MDA-5. Consequently, this combined treatment of tumor cells triggers a robust synergistic amplification of dsRNA-induced inflammation and immunogenicity. Importantly, in diverse coculture models, our approach elicited potent activation of autologous CD8^+^ TILs directed against both TAAs and tumor-specific antigens, as well as improved DC maturation. Using publicly available melanoma datasets, we confirmed the clinical relevance of these findings, as an antiviral gene signature enhanced by BETi was negatively correlated with tumor-intrinsic immune resistance and positively associated with immune infiltration and patient survival.

Based on our data, we postulate that BETi reprograms tumors by suppressing a range of (IFN-inducible) negative regulators of antiviral signaling, thereby rendering them highly sensitive to subsequent stimulation with RLR agonists in the absence of these tumor-intrinsic intracellular “immunologic brakes.” This interpretation aligns with the established notion that BET proteins function as transcriptional activators of tumor-expressed immunosuppressive factors, with PD-L1 serving as the best-characterized example.

Although classical BETi like JQ1 and I-BET151 can sensitize tumor cells to dsRNA-induced signaling, dBET1 failed to do so in our models. dBET1 is a PROTAC degrader that links JQ1 to a cereblon ligand, inducing proteasomal degradation of BET proteins. In contrast, JQ1 and I-BET151 competitively inhibit the binding of BET proteins to acetyllysine residues on chromatin (35). These mechanistic differences have been reported to yield divergent biological effects. For example, BRD4 not only drives MYC transcription but also promotes phosphorylation-dependent MYC degradation to maintain homeostasis (36). Consequently, competitive BETi can suppress MYC transcription without destabilizing MYC protein, whereas dBET1-like degraders can reduce MYC transcription while paradoxically increasing MYC protein levels. Surprisingly, MYC depletion did not seem to be a key driver of the effects revealed by our study, as direct pharmacologic MYC inhibition did not recapitulate the sensitization observed upon JQ1 pretreatment. Thus, although it is still unclear what drives the differential response to dsRNA in tumor cells pretreated with JQ1, I-BET151, or dBET1, our data suggest that competitive inhibition, rather than degradation, of BET proteins is critical for the sensitization of tumor cells to dsRNA-induced signaling and does not require MYC depletion.

The precise molecular determinants mediating tumor sensitivity to RLR agonists after BET inhibition remain incompletely defined. Our study suggests that BETi might sensitize tumor cells by suppressing negative regulators of MAVS signaling, including ADAR1 and NLRX1. However, as our transcriptomic analysis revealed that multiple negative regulators of viral sensing pathways are downregulated by JQ1, the enhanced tumor cell sensitivity to dsRNA induced by BETi more likely reflects a cumulative effect of broad transcriptional rewiring rather than dependence on a single molecule.

We propose that BET inhibition serves as a permissive switch in tumor cells that allows RLR agonists to trigger a broad antiviral program, characterized by enhanced expression of molecules that promote antitumor T-cell responses. Using human *in vitro* models, we assessed key determinants of tumor immunogenicity to CD8^+^ T cells, including HLA-I antigen processing and presentation, as well as soluble mediators such as IFNβ, IFNλ1, CXCL10, and CXCL11. Although the HLA-I APM is a well-established determinant of tumor immunogenicity to CD8^+^ T cells, accumulating evidence indicates the importance of these soluble mediators in recruiting and activating antitumoral CD8^+^ T cells (37). Therefore, further studies are needed to delineate the direct and indirect mechanisms by which BET inhibition combined with RLR stimulation might drive antitumor immune responses *in vivo*.

Although conserved RIG-I and IFN signaling are critical for mediating responses to immunotherapy, chronic IFN signaling has also been shown to drive immune escape (38). For example, in melanoma, chronic inflammation can result in tumor cell dedifferentiation and the loss of T cell–recognized antigens (39). Here, we show that BETi amplifies antigen presentation and activation of neoantigen-specific T cells while preserving MART-1–specific T-cell recognition despite pIC-induced dedifferentiation. This indicates that BET inhibition can enhance RLR-induced immunogenicity and mitigate nonredundant tumor-intrinsic immune escape mechanisms.

BETi were initially developed clinically for their antiproliferative and proapoptotic effects, which were largely attributed to their ability to target MYC (40). However, early-phase clinical trials yielded limited benefits, partly due to dose-limiting toxicities (41). Our study suggests that BETi could be repurposed as immunomodulatory sensitizers that can be used at subapoptotic doses to reprogram tumor-intrinsic antiviral pathways and prime tumor cells for subsequent stimulation with an RLR agonist.

Beyond indirectly promoting immune responses by enhancing tumor immunogenicity, BETi have also been shown to reverse T-cell and NK cell exhaustion (42, 43). Given the pleiotropic and cell type–dependent effects of BETi, defining optimal timing, dosing, and combination strategies will be essential to fully harness their therapeutic potential. Moreover, further *in vivo* studies are needed to evaluate the feasibility, safety, and therapeutic efficacy of combining BETi with RLR agonists.

In summary, this study identifies a previously unrecognized role for BET proteins in driving tumor-intrinsic immune resistance mechanisms and establishes a rationale for using BET inhibition to sensitize tumors to RLR agonist-based treatments. The broad and synergistic effects of this combined treatment create highly immunogenic tumor cells, providing a basis for their use in the antigen-agnostic activation of tumor-specific T cells in *ex vivo* and *in vivo* immunotherapy applications. Further research in relevant preclinical models will be important to address this and to evaluate whether such immunotherapies could affect clinical outcomes and immune-resistant disease.

## Supplementary Material

Supplementary Figure S1 Gating strategies used in flow cytometry for tumor cells, T cell and DC stainings.

Supplementary Figure S2 Differentiation status of melanoma cell lines.

Supplementary Figure S3 JQ1 pre-treatment sensitizes BRAF-resistant A375VR4 cells to poly(I:C)-induced HLA-I.

Supplementary Figure S4 Effect of I-BET151 and dBET1 on sensitizing to poly(I:C)-induced HLA-I.

Supplementary Figure S5 Enriched gene programs from JQ1 and/or poly(I:C) treatment derived RNA sequencing analysis (related to figure 2).

Supplementary Figure S6 Gene programs suppressed by JQ1 and/or JQ1 + IFNγ treatment (related to figure 3).

Supplementary Figure S7 Role of MYC in observed effect after BETi treatment.

Supplementary Figure S8 BETi treatment enhances MAVS expression.

Supplementary Figure S9 BETi selectively enhances RLR-induced HLA-I in cancer cells.

Supplementary Figure S10 Effect of JQ1 and/or poly(I:C) treatment on CD8+ vs CD4+ T cell activation.

Supplementary Figure S11 Effect of JQ1 and/or poly(I:C) treatment on melanoma differentiation.

Supplementary Figure S12 JQ1 pre-treatment sensitizes BRAF-resistant A375VR4 cells to poly(I:C)-induced cell surface calreticulin expression.

Supplementary Figure S13 Supplementary data from transcriptomic analysis of TGCA melanoma cohort.

Supplementary Tables S1-S6 Supplementary Tables S1-S6.

## Data Availability

Sequencing data are available in the Sequence Read Archive (PRJNA1357665). Code from this study is publicly available at https://github.com/mgerault/BETdsRNA.Baldran. Additional data are available upon reasonable request.

## References

[1] Hamdan F , Cerullo V. Cancer immunotherapies: a hope for the uncurable? Front Mol Med 2023;3:1140977. 10.3389/fmmed.2023.1140977. PMC11285639 39086690

[2] Wang Y , Jasinski-Bergner S, Wickenhauser C, Seliger B. Cancer immunology: immune escape of tumors—expression and regulation of HLA class I molecules and its role in immunotherapies. Adv Anat Pathol 2023;30:148–59. 10.1097/PAP.0000000000000389. 36517481

[3] Zaretsky JM , Garcia-Diaz A, Shin DS, Escuin-Ordinas H, Hugo W, Hu-Lieskovan S, . Mutations associated with acquired resistance to PD-1 blockade in melanoma. N Engl J Med 2016;375:819–29. 10.1056/NEJMoa1604958. PMC5007206 27433843

[4] Pang L , Zhou F, Liu Y, Ali H, Khan F, Heimberger AB, . Epigenetic regulation of tumor immunity. J Clin Investig 2024;134:e178540. 10.1172/JCI178540. PMC11178542 39133578

[5] Delmore JE , Issa GC, Lemieux ME, Rahl PB, Shi J, Jacobs HM, . BET bromodomain inhibition as a therapeutic strategy to target c-myc. Cell 2011;146:904–17. 10.1016/j.cell.2011.08.017. PMC3187920 21889194

[6] Hogg SJ , Vervoort SJ, Deswal S, Ott CJ, Li J, Cluse LA, . BET-bromodomain inhibitors engage the host immune system and regulate expression of the immune checkpoint ligand PD-L1. Cell Rep 2017;18:2162–74. 10.1016/j.celrep.2017.02.011. PMC5340981 28249162

[7] Zhang M , Wang G, Ma Z, Xiong G, Wang W, Huang Z, . BET inhibition triggers antitumor immunity by enhancing MHC class I expression in head and neck squamous cell carcinoma. Mol Ther 2022;30:3394–413. 10.1016/j.ymthe.2022.07.022. PMC9637808 35923111

[8] Streicher F , Jouvenet N. Stimulation of innate immunity by host and viral RNAs. Trends Immunol 2019;40:1134–48. 10.1016/j.it.2019.10.009. 31735513

[9] Such L , Zhao F, Liu D, Thier B, Le-Trilling VTK, Sucker A, . Targeting the innate immunoreceptor RIG-I overcomes melanoma-intrinsic resistance to T cell immunotherapy. J Clin Invest 2020;130:4266–81. 10.1172/JCI131572. PMC7410049 32427578

[10] Wickström S , Lövgren T. Expansion of tumor-infiltrating lymphocytes from melanoma tumors. Methods Mol Biol 2019;1913:105–18. 10.1007/978-1-4939-8979-9_7. 30666601

[11] Wickström SL , Lövgren T, Volkmar M, Reinhold B, Duke-Cohan JS, Hartmann L, . Cancer neoepitopes for immunotherapy: discordance between tumor-infiltrating T cell reactivity and tumor MHC peptidome display. Front Immunol 2019;10:2766. 10.3389/fimmu.2019.02766. PMC6918724 31921104

[12] Cancer Genome Atlas Network . Genomic classification of cutaneous melanoma. Cell 2015;161:1681–96. 10.1016/j.cell.2015.05.044. PMC4580370 26091043

[13] Duewell P , Beller E, Kirchleitner SV, Adunka T, Bourhis H, Siveke J, . Targeted activation of melanoma differentiation-associated protein 5 (MDA5) for immunotherapy of pancreatic carcinoma. Oncoimmunology 2015;4:e1029698. 10.1080/2162402X.2015.1029698. PMC4589065 26504669

[14] Zhu H , Bengsch F, Svoronos N, Rutkowski MR, Bitler BG, Allegrezza MJ, . BET bromodomain inhibition promotes anti-tumor immunity by suppressing PD-L1 expression. Cell Rep 2016;16:2829–37. 10.1016/j.celrep.2016.08.032. PMC5177024 27626654

[15] Jiang Y , Zhang H, Wang J, Chen J, Guo Z, Liu Y, . Exploiting RIG-I-like receptor pathway for cancer immunotherapy. J Hematol Oncol 2023;16:8. 10.1186/s13045-023-01405-9. PMC9906624 36755342

[16] Zou Z , Huang B, Wu X, Zhang H, Qi J, Bradner J, . Brd4 maintains constitutively active NF-κB in cancer cells by binding to acetylated RelA. Oncogene 2014;33:2395–404. 10.1038/onc.2013.179. PMC3913736 23686307

[17] Hugo W , Zaretsky JM, Sun L, Song C, Moreno BH, Hu-Lieskovan S, . Genomic and transcriptomic features of response to anti-PD-1 therapy in metastatic melanoma. Cell 2016;165:35–44. 10.1016/j.cell.2016.02.065. PMC4808437 26997480

[18] Das A , Dinh PX, Pattnaik AK. Trim21 regulates Nmi-IFI35 complex-mediated inhibition of innate antiviral response. Virology 2015;485:383–92. 10.1016/j.virol.2015.08.013. 26342464

[19] Mérour E , Jami R, Lamoureux A, Bernard J, Brémont M, Biacchesi S. A20 (tnfaip3) is a negative feedback regulator of RIG-I-Mediated IFN induction in teleost. Fish Shellfish Immunol 2019;84:857–64. 10.1016/j.fsi.2018.10.082. 30385247

[20] Lee K , Lin C-C, Servetto A, Bae J, Kandagatla V, Ye D, . Epigenetic repression of STING by MYC promotes immune evasion and resistance to immune checkpoint inhibitors in triple-negative breast cancer. Cancer Immunol Res 2022;10:829–43. 10.1158/2326-6066.CIR-21-0826. PMC9250627 35561311

[21] Wu M , Pei Z, Long G, Chen H, Jia Z, Xia W. Mitochondrial antiviral signaling protein: a potential therapeutic target in renal disease. Front Immunol 2023;14:1266461. 10.3389/fimmu.2023.1266461. PMC10602740 37901251

[22] Kalbasi A , Tariveranmoshabad M, Hakimi K, Kremer S, Campbell KM, Funes JM, . Uncoupling interferon signaling and antigen presentation to overcome immunotherapy resistance due to JAK1 loss in melanoma. Sci Transl Med 2020;12:eabb0152. 10.1126/scitranslmed.abb0152. PMC8053376 33055240

[23] Thier B , Paschen A. Innate RIG-I signaling restores antigen presentation in tumors and overcomes T cell resistance. Cell Stress 2021;5:26–8. 10.15698/cst2021.02.242. PMC7841847 33554047

[24] Feola S , Chiaro J, Martins B, Cerullo V. Uncovering the tumor antigen landscape: what to know about the discovery process. Cancers 2020;12:1660. 10.3390/cancers12061660. PMC7352969 32585818

[25] Thier B , Zhao F, Stupia S, Brüggemann A, Koch J, Schulze N, . Innate immune receptor signaling induces transient melanoma dedifferentiation while preserving immunogenicity. J Immunother Cancer 2022;10:e003863. 10.1136/jitc-2021-003863. PMC9196182 35697379

[26] Lambing S , Tan YP, Vasileiadou P, Holdenrieder S, Müller P, Hagen C, . RIG-I immunotherapy overcomes radioresistance in p53-positive malignant melanoma. J Mol Cell Biol 2023;15:mjad001. 10.1093/jmcb/mjad001. PMC10394996 36626927

[27] Duewell P , Steger A, Lohr H, Bourhis H, Hoelz H, Kirchleitner SV, . RIG-I-like helicases induce immunogenic cell death of pancreatic cancer cells and sensitize tumors toward killing by CD8^+^ T cells. Cell Death Differ 2014;21:1825–37. 10.1038/cdd.2014.96. PMC4227156 25012502

[28] Elion DL , Jacobson ME, Hicks DJ, Rahman B, Sanchez V, Gonzalez-Ericsson PI, . Therapeutically active RIG-I agonist induces immunogenic tumor cell killing in breast cancers. Cancer Res 2018;78:6183–95. 10.1158/0008-5472.CAN-18-0730. 30224377

[29] Roussot N , Ghiringhelli F, Rébé C. Tumor immunogenic cell death as a mediator of intratumor CD8 T-cell recruitment. Cells 2022;11:3672. 10.3390/cells11223672. PMC9688834 36429101

[30] Tesniere A , Panaretakis T, Kepp O, Apetoh L, Ghiringhelli F, Zitvogel L, . Molecular characteristics of immunogenic cancer cell death. Cell Death Differ 2008;15:3–12. 10.1038/sj.cdd.4402269. 18007663

[31] Lei X , Khatri I, de Wit T, de Rink I, Nieuwland M, Kerkhoven R, . CD4^+^ helper T cells endow cDC1 with cancer-impeding functions in the human tumor micro-environment. Nat Commun 2023;14:217. 10.1038/s41467-022-35615-5. PMC9839676 36639382

[32] Gerhard GM , Bill R, Messemaker M, Klein AM, Pittet MJ. Tumor-infiltrating dendritic cell states are conserved across solid human cancers. J Exp Med 2021;218:e20200264. 10.1084/jem.20200264. PMC7754678 33601412

[33] Luca BA , Steen CB, Matusiak M, Azizi A, Varma S, Zhu C, . Atlas of clinically distinct cell states and ecosystems across human solid tumors. Cell 2021;184:5482–96.e28. 10.1016/j.cell.2021.09.014. PMC8526411 34597583

[34] Jerby-Arnon L , Shah P, Cuoco MS, Rodman C, Su M-J, Melms JC, . A cancer cell program promotes T cell exclusion and resistance to checkpoint blockade. Cell 2018;175:984–97.e24. 10.1016/j.cell.2018.09.006. PMC6410377 30388455

[35] Winter GE , Buckley DL, Paulk J, Roberts JM, Souza A, Dhe-Paganon S, . DRUG DEVELOPMENT. Phthalimide conjugation as a strategy for in vivo target protein degradation. Science 2015;348:1376–81. 10.1126/science.aab1433. PMC4937790 25999370

[36] Devaiah BN , Mu J, Akman B, Uppal S, Weissman JD, Cheng D, . MYC protein stability is negatively regulated by BRD4. Proc Natl Acad Sci U S A 2020;117:13457–67. 10.1073/pnas.1919507117. PMC7306749 32482868

[37] House IG , Savas P, Lai J, Chen AXY, Oliver AJ, Teo ZL, . Macrophage-derived CXCL9 and CXCL10 are required for antitumor immune responses following immune checkpoint blockade. Clin Cancer Res 2020;26:487–504. 10.1158/1078-0432.CCR-19-1868. 31636098

[38] Zhang X , Wang S, Zhu Y, Zhang M, Zhao Y, Yan Z, . Double-edged effects of interferons on the regulation of cancer-immunity cycle. Oncoimmunology 2021;10:1929005. 10.1080/2162402X.2021.1929005. PMC8253121 34262796

[39] Kim YJ , Sheu KM, Tsoi J, Abril-Rodriguez G, Medina E, Grasso CS, . Melanoma dedifferentiation induced by interferon-gamma epigenetic remodeling in response to anti-PD-1 therapy. J Clin Invest 2021;131:e145859. 10.1172/JCI145859. PMC8203459 33914706

[40] Shorstova T , Foulkes WD, Witcher M. Achieving clinical success with BET inhibitors as anti-cancer agents. Br J Cancer 2021;124:1478–90. 10.1038/s41416-021-01321-0. PMC8076232 33723398

[41] Wang Z-Q , Zhang Z-C, Wu Y-Y, Pi Y-N, Lou S-H, Liu T-B, . Bromodomain and extraterminal (BET) proteins: biological functions, diseases, and targeted therapy. Signal Transduct Target Ther 2023;8:420. 10.1038/s41392-023-01647-6. PMC10625992 37926722

[42] Reggiani F , Talarico G, Gobbi G, Sauta E, Torricelli F, Manicardi V, . BET inhibitors drive Natural Killer activation in non-small cell lung cancer via BRD4 and SMAD3. Nat Commun 2024;15:2567. 10.1038/s41467-024-46778-8. PMC10960013 38519469

[43] Romine KA , MacPherson K, Cho H, Kosaka Y, Flynn PA, Byrd KH, . BET inhibitors rescue anti-PD1 resistance by enhancing TCF7 accessibility in leukemia-derived terminally exhausted CD8^+^ T cells. Leukemia 2023;37:580–92. 10.1038/s41375-023-01808-0. PMC9991923 36681742

